# NLRC3 Delays the Progression of AD in APP/PS1 Mice via Inhibiting PI3K Activation

**DOI:** 10.1155/2020/5328031

**Published:** 2020-12-24

**Authors:** Lihuang Zha, Zaixin Yu, Jian Fang, Li Zhou, Wen Guo, Jun Zhou

**Affiliations:** ^1^Department of Cardiology, Xiangya Hospital, Central South University, Changsha, Hunan 410008, China; ^2^Department of Pathology, Xiangya Hospital, Central South University, Changsha, Hunan 410008, China; ^3^Institute of Physical Education, Hunan Normal University, Changsha, China 410008; ^4^Medical Science Research Center, Xiangya Hospital, Central South University, Changsha, Hunan 410008, China

## Abstract

NLRC3 inhibits inflammatory responses. Neuroinflammation induces and accelerates the onset of Alzheimer's disease (AD). This study is aimed at investigating whether NLRC3 plays a role in neuroinflammation, A*β* accumulation, and neuroprotection in AD mice. 12-month-old APP/PS1 transgenic and C57 mice were used for studies in vivo. In vitro, organotypic hippocampal slices were cultured. We found that the expression of NLRC3 was downregulated in the brain tissues of APP/PS1 mice. Mice in the APP/PS1 group had a significant attenuation of learning and memory ability compared to the control group, and the ability was improved in APP/PS1 + LV-NLRC3 mice. The expressions of 6E10, GFAP, Iba1, and PI3K in the hippocampus and brains of APP/PS1 mice were significantly higher than those of the control group, while the expressions of NeuN were lower than that of the control group. With the overexpression of NLRC3 in the APP/PS1 + LV-NLRC3 group, the expressions of 6e10, GFAP, Iba1, and PI3K were significantly lower, while the expression of NeuN was significantly higher compared to the APP/PS1 group. NLRC3 colocalized with NeuN. PI3K activation with 740YP increased the expression of GFAP and Iba-1 in the hippocampus with the exogenous NLRC3 protein. We conclude that NLRC3 may play an important role in the development and progression of AD. Downregulation of NLRC3 can lead to the activation of PI3K, resulting in abnormal plaque deposition, glial cell activation, and neuron loss during AD. NLRC3 delays the progression of AD in APP/PS1 mice via inhibiting PI3K activation.

## 1. Introduction

Alzheimer's disease (AD) is a progressive neurodegenerative disorder and the most common cause of age-related dementia. It involves deterioration of the memory and cognitive function that finally results in death. As the World Alzheimer Report (Prince, The Global Impact of Dementia: an Analysis of Prevalence, Incidence, Cost and Trends, 2015), there are more than 46.8 million people worldwide suffering from AD, and the incidence is still increasing. This presents an enormous threat especially to the physical and mental health of the elderly population [1].

The main neuropathological features of AD are formation of senile plaques composited by amyloid beta (A*β*) peptide fibrils and dystrophic neuritis, neurofibrillary tangles (NFTs) formed of abnormal phosphorylated tau protein, neuronal loss, and cerebrovascular amyloidosis. Both A*β* aggregation and activation of glial cells are supposed essential in the pathological process of AD [2, 3]. Studies have shown that the increase of astrocyte and microglia production in the human brain has a linear relationship with the progress of disease. In AD patients with mild cognitive impairment, the production of glial cells is considered to be an early phenomenon in the development of AD [4–6]. Currently, there is no particularly effective treatment, and the pathogenesis of AD is still unclear [7]. Neuroinflammation mediated by gliocyte activation and neuron loss are identified as two major features commonly seen in AD [8–10]. Microglia are resident innate immune cells which play important roles in regulating neurotoxicity caused by the inflammatory response in the central nervous system (CNS). In adult humans or mice, the density of microglia varies in different brain regions, and their concentration is the highest in the hippocampus. Lipopolysaccharide (LPS) or A*β* can activate microglia, leading to the overexpression of inflammatory cytokines such as IL-6, IL-1*β*, and reactive oxygen species (ROS). The overexpression of various inflammatory cytokines contributes to nerve injury, which leads to the occurrence and development of AD [11]. Therefore, antineuroinflammation via inhibition of microglial overactivation is regarded as a promising new strategy for preventing AD [12, 13]. Astrocytes, one the most important gliocyte, have supportive and protective functions to neurons in the brain. Astrocytes dynamically regulate the signal pathway, regulate the plasticity of neurons and synapses, and provide nutrition and metabolism support for neurons in the brain. Local or chronic inflammation of the brain is an important pathophysiological mechanism in AD, which is characterized by activated microglia and astrocytes around amyloid plaques, and neurofibrillary tangles formation [14, 15].

Nucleotide-oligomerization domain-like receptors (NLRs), which belong to a large family of cytoplasmic sensors, are pattern recognition receptors associated with immunity and inflammation in response to pathogen and damage-associated molecular patterns. Dysregulation of their functions is found in several diseases including various cancers, metabolic diseases, autoimmune disorders, and inflammatory diseases [16, 17]. NLRC3 (also known as CLR16.2 or NOD3), a newly discovered and incompletely characterized member of the NLR family, has a typical nucleotide binding domain-leucine-rich repeat configuration at its central and C-terminal domains and a caspase activation and recruitment domain (CARD) 3 at the amino terminal [18]. Previous studies have reported that NLRC3 negatively regulates NLR-mediated inflammatory responses by competing to bind inflammasome components, inhibiting the activation of caspase-1 and reducing the release of IL-1*β* [19]. Recently, it has been demonstrated that NLRC3 inhibition may trigger proinflammatory cytokine production by activating phosphoinositide 3-kinase (PI3K) and its downstream transcription factors [20–22]. Several studies have shown that abnormal activation of PI3K/AKT signaling in neurons plays an crucial role in the occurrence and development of AD [23, 24].

However, the pathophysiological role of NLRC3 in AD remains largely unclear. In both in vitro and in vivo studies, little information is available on the neuroinflammatory and neuroprotective effects of NLRC3. In vivo, numerous clinical imaging and neuropathology studies suggest that gliocytes form the basis of the brain's immune system, and activated gliocytes play prominent roles in the pathogenesis of AD [25]. In NLRC3-like−/− zebrafish, primitive macrophages initiate a systemic inflammation with increased and actively aggregated proinflammatory cytokines, highlighting an undefined potential function of NLRC3 in the development of AD [26].

In this study, we constructed lentiviral vectors containing NLRC3 through intracerebroventricular injection in 12-month-old APPswe/PS1-dE9 (amyloid precursor protein/presenilin protein 1 (APP/PS1)) double transgenic model mice which represents mid-term of AD when animals develop A*β* formation, show pathological changes in brain tissue and structure, and exhibit learning and memory function obstacles. Based on the theory above, we speculate that NLRC3 may inhibit the A*β*-induced gliocyte activation and release of inflammatory mediators via inhibiting the activation of PI3K and its downstream signals. NLRC3 is a newly discovered member of the NOD family. There are few studies of NLRC3 in AD that have been conducted. In this study, we first evaluate the expression of NLRC3 in brain tissues of the model mice and then examine the effects of NLRC3 on learning and memory ability, A*β* deposition, activation of glial cells, and neurons degeneration in APP/PS1 mice, which further explored its mechanisms. Therefore, the aim of this study is to explore the neuroprotective effect of NLRC3 in A*β*-induced activated gliocytes and the early therapeutic effect of NLRC3 in AD mice. We wonder whether NLRC3 plays a neuroprotective role through inhibiting the A*β*-induced gliocytes activation and release of inflammatory mediators via PI3K and its downstream signals.

## 2. Materials and Methods

### 2.1. Animals and Ethics Statement

APP/PS1 double transgenic mice were bought from the Model Animal Research Center of Nanjing University [animal license number: SCXK (su) 2012–0007, stock number: 0014406]. All time points referred to herein indicate the number of days after transferation of APP/PS1. The new transgenic AD APP/PS1 mouse model was obtained by breeding APP/PS1 AD mice, and the genotypes were confirmed by PCR of mouse tail genomic DNA using the 2∗EasyTaq PCR SuperMix (Trans Technology, Lot:), according to the manufacturer's instructions (Figure S1). All the experimental procedures were approved by the Animal Laboratory Administrative Center and the Institutional Ethics Committee at Xiangya Medical University and also in accordance with the National Institutes of Health guidelines. Specific primers for the APP gene contained within the Sangon Biotech (Shanghai) designed (forward Neo5′; 5′-GACTGACCACTCGACCAGGTTCTG-3′ and reverse Neo3′; 5′-CTTGTAAGTTGGATTCTCATATCCG-3′). Also, specific primers for the PS1 gene contained within the Sangon biotech (Shanghai) designed (forward Neo5′; 5′-AATAGAGAACGGCAGGAGCA-3′ and reverse Neo3′; 5′-GTGGATAACCCCTCCCCCAGCCTAGACC-3). After PCR reactions, the amplified products were separated in agarose gels and analyzed. C57/BL6 as wild type was purchased from the Experimental Animal Center of Jingda [animal license number: SCXK (xiang) 2013–0004]. There were 4 groups including C57/BL6 as the wild type (control) group, APP/PS1 (model) group, APP/PS1 + LVCON307 group, and APP/PS1 + LV-NLRC3 group. Thirty 12-month-old APP/PS1 transgenic mice were randomized into three groups as the model group, APP/PS1 + LVCON307, and APP/PS1 + LV-NLRC3 group. Ten 12-month-old wild type C57 mice were chosen as the control group. Mice in the APP/PS1 + LVCON307 and APP/PS1 + LV-NLRC3 group were injected with LVCON307 or LV-NLRC3 through intracerebroventricular injection (4 *μ*l, 10^7^ virus particles (VPs)/kg). Mice in the control group and model group were administrated with the same volume of normal saline. The study animals were housed in barrier facilities (Experimental Animal Center, Xiangya Medical School of Central South University) at a constant temperature of 22°C and at 40-60% humidity levels, under a 12 : 12 h light–dark cycle (light 7 : 00–19 : 00); ), with ad libitum food and water (Figure S2).

### 2.2. Lentiviral Vectors (LV)

In this study, the construction and titer determination of LV-NLRC3 and negative control lentivirus (LVCON307) were constructed and purified by GeneChem Biomedical Co. Ltd. (Shanghai, China). The titers were 5E+8 and 6E+8 transducing units (TU)/ml.

### 2.3. Intracerebroventricular Injections

Mice were anesthetized by intraperitoneal injection of ketamine and intracerebroventricular injection with microinjector. The bregma point was fully exposed by cutting an incision about 0.5 cm long along the median line of the head at the beginning of the operation. At 1.5 mm posterior to the point and 1 mm lateral to the right, the microinjector was used to penetrate the skull vertically at a depth of 3 mm and retained for 5-10 minutes. 4 *μ*l of LV-NLRC3 or LVCON307 was then slowly injected at the rate of 1 *μ*l/min, and the needle retained in position for a further 5-10 minutes. The needle was then withdrawn, and the skin wound closed with sutures. After 6 months, the mice were decapitated and executed. The brains were quickly removed, placed on ice, and sagittally sectioned along the median line.

### 2.4. Morris Water Maze and Behavior Test

The Morris water maze was used to evaluate the spatial learning and memory (SLM) function of mice at age 18 month in each group, according to the protocols of van Praag et al. (1999) and Akers et al. (2014). Mice were housed and habituated for 2 h in behavioral testing rooms before tests. All tests were conducted on consecutive days in a dimly illuminated room with standard conditions of temperature and noise insulation. All behavioral apparatus were cleaned with 75% ethanol and dried between each animal. Behavioral outcomes were recorded by two trained assistants who were blinded to the information of each mouse. The apparatus consisted of a circular tub (120 cm in diameter, 50 cm in height) with a black inner wall and a transparent platform (10 cm diameter) submerged 1 cm below the water surface, which was painted with distinct geometric cues. The water was maintained at temperature of 24 ± 1°C. Forty mice underwent four trials per day in the learning stage. Each mouse was placed into the water and randomly started from each of four different locations facing the pool wall in the trial. The trial was terminated, and the latency was recorded when the mouse found the platform within 90 s. Otherwise, the trial was terminated, and the mouse was gently guided to the platform. On day 5, a probe trial was conducted to estimate the memory function of each mouse. After the platform was removed, the mouse was put into the pool as before to evaluate its memory ability. The swim paths were recorded with an overhead video camera and tracked with automated software (San Diego Instruments, San Diego, CA, USA). As the time to reach the platform during water maze training, number of the times the target area (former platform) was crossed, and time spent in each quadrant of the tub was recorded during the probe trial.

### 2.5. Histology

After the Morris water maze test, five mice from each group were deeply anesthetized (300 mg/kg, i.p. 10% chloral hydrate in DDW) and were perfused with ice-cold normal saline (50 ml) to remove blood from the vasculature and then with 4% paraformaldehyde in phosphate buffered saline(50 ml). Their brains were removed and incubated overnight in 4% paraformaldehyde, and then half of each brain was dehydrated in 30% sucrose in PBS for frozen sections, and the other half was treated with routine fixation, dehydration, and paraffin embedding for paraffin sections. Another five mice from each group were deeply anesthetized and perfused with ice-cold normal saline (50 ml), and then their brain samples were harvested and stored at -80°C for real-time PCR and western blot analysis.

### 2.6. Organotypic Hippocampal Slice Culture

Prepare surgical equipment: brushes, scissors (big and small), plastic pipette (thin, middle, and short), blades, plastic plates, and curved dissecting forceps and plastic knifes (long). Make sure to keep everything sterilized. Sterilize the surface of the super clean workbench with alcohol and turn the ultraviolet light on, sterilizing for two hours. Use a container of ice to place the cutting medium. The composition of the cutting medium is (pH = 7.2) Earle's MEM (cat.61100-06, Lot.74 k4064 Gibco), HEPES 5.95 g, Tri-base 1.21 g, glucose 1.8 g, and MgCl2 6 ml (all reagents from Gibco or Sigma). Get packs of cell well dishes and place inside each of the wells an insert of Millipore. Add to the outside edge of the well 1 mL of tissue medium. Put dishes into the incubator.

Add 1 ml tissue culture medium to each well of 6-well plate, and inserts of Millipore were then placed into the wells where the tissue culture medium had been added. The composition of the culture medium is (pH = 7.25, in 1 L) BME (B 9638), EBSS (E7510), NaCI (S5886) 1.167 g, NaHCO3 (S 5761) 0.42 g, ascorbic acid (A 4544), glucose (G 7021), HEPES (free acid H3375) 6.36 g, CaCl_2_·2H_2_O (C 7903) 0.0293 g, MgS04 (M 2643) 0.203 g, glutamine (G 7029), 0.39 g, insulin (2 mg/ml stock I4011) 0.667 ml, penicillin (P 3032) 24.8 mg, and 20% horse serum (all reagents from Gibco or Sigma).Put dishes into the incubator. Prepare the chopper (LEICA VT1200S, VIBRATOME LINE), cut head of pups (postnatal 6-9 days) quickly, and remove the skull and membrane till you get to the brain. Put the brain in cutting medium with paper and slice into two pieces and get the hippocampus. Use a brush to gently move the separated pieces of the hippocampus with cutting medium onto chopping board. Cut the hippocampus into 400 micron slices and gently place these sections in cutting medium. Get the spare 6-well plates containing the culture medium from the incubator. Place 2-5 pieces of dorsal hippocampal at the approximal coronal section in each pup on the Millipore inserts of each well. Excess cutting medium should be sucked. Place plate in the incubator and label (name, date). After 2-3 hours, use sucker to remove old medium of the bottom in each well and add new culture medium. Feed the slice culture tissue each other days about 10 days. Treatment slices culture tissue about 6-10 days. The NLRC3 protein was used for 48 hours, A*β* was used 36 hours before tissue harvested.

### 2.7. Immunohistochemistry, Immunofluorescent, and Confocal Microscopy

Immunohistochemistry was performed to investigate the expression of A*β*, NLRC3, and glial fibrillary acidic protein (GFAP) in the mice brains. Briefly, brains sections were dewaxed and rehydrated in decreasing concentrations of ethanol. Then, endogenous peroxidase of the sections was blocked with 3% H_2_O_2_ at room temperature for 10 minutes. Slides were washed with PBS several times and blocked in 2% BSA with 0.3% Triton X-100 for 40 min at 37°C. Thereafter, the sections were incubated with primary antibodies (GFAP 1 : 200 Millipore, Billerica, MA, USA; 6E10 1 : 1000 Thermo Fisher Scientific; NLRC3 1 : 100 abnova, Taiwan, China) overnight at 4°C. They were then rinsed in PBS and incubated with biotinylated secondary antibody (Vector, 1 : 200) and streptavidin-horseradish peroxidase (Jackson, Immunoresearch, West Grove, PA, USA) for 1 h at 37°C and then rinsed for another 3 min three times with PBS before reaction with diamino-benzidine (DAB) (Vector) solution. The sections were observed under a microscope. Nissl staining was performed to quantify the neuronal density in the sections.

For immunofluorescent staining, the sections were boiled in citric acid buffer (pH 6.0) for 20 min in a bain marie oven. After cooling, they were treated with 0.3% Triton X-100 and 2% BSA for 1 h at room temperature. The sections were then incubated overnight at 4°C with a primary antibody (neuron-specific nuclear protein (NeuN) 1 : 200 CST, USA; NLRC3 1 : 100 abnova, Taiwan, China) and then with a secondary antibody (1 : 300 Alexa-Fluor-488-conjugated goat anti-mouse IgG2a antibody, Life Technologies, catalog number A21131; 1 : 300 Alexa-Fluor-555-conjugated goat anti-rabbit IgG1 antibody, Life Technologies, catalog number A31572) in PBS containing 1% BSA at room temperature for 2 h. The sections were mounted onto slides, embedded with SlowFade® Gold (Invitrogen), and covered with a coverslip.

A*β* plaques in the brains were visualized using thioflavin-S fluorescence (ThS) staining. ThS was dissolved in 50% of ethanol at 500 mM, and brain sections were stained for 7 min. As a differentiation step to remove a nonspecific binding of the dye, a slide was soaked into 100%, 95%, and 90% ethanol solutions for 10 sec each and then moved into PBS.

### 2.8. Quantitative Real-Time PCR

The level of NLRC3 mRNA was detected using real-time qPCR kits by technicians who were blinded to the experimental groups. Total RNA was extracted from the frontal cortex and hippocampus using the Trizol Plus RNA Purification System (Ambion, Invitrogen) according to the manufacturer's instructions. RNA was quantified using the BioSpec Nano spectrophotometer (Shimadzu), and cDNA was reverse transcribed using the cDNA Synthesis kit (Thermo scientific, Lot 00285583) according to the manufacturer's instructions. Quantitative real-time PCR was performed using the UltraSYBR Mixture (With ROX1) (CEBIO, CW2601) with the following cycling parameters: 95°C for 10 minutes followed by 38 cycles of 95°C for 10 seconds, 60°C for 30 seconds, 72°C for 32 seconds, and amplification dissociation (95°C for 15 seconds, 60°C for 1 minute, 95°C for 15 seconds, 60°C for 15 seconds, increasing at 0.5°C/cycle until 95°C was reached). All gene expression data were normalized to the *β*-actin expression. All reactions were performed in triplicate. Gene expression results were calculated using the delta-delta cycle threshold (two delta-delta CT) method (Livak and Schmittgen, 2001). The two delta-delta CT method was used to determine mean fold changes in the gene expression between the control and target genes.

### 2.9. Western Blot

Brain tissues were lysed in RIPA buffer containing phosphatase inhibitor and complete protease inhibitor cocktail. Aliquots of brain lysates were separated on SDS-PAGE, transferred to membranes, and immunoblotted with primary antibodies, then horseradish peroxidase-conjugated secondary antibody (1 : 1000). Bands were revealed by use of an enhanced chemiluminescence (ECL) system (Thermo Scientific, Rockford IL), and density was quantified by the use of Imagequant 5.2 (Healthcare Bio-Sciences, Philadelphia, PA). The primary Abs were used as follows: Iba-1 and NeuN (1 : 1000 dilution, CST, USA), GFAP (1 : 1000 dilution; Millipore, USA), NLRC3(1 : 1000 dilution; abnova), PI3K (1 : 500 dilution, CST, USA), *β*-actin, and GAPDH (Proteintech, Chicago, USA). Secondary horseradish peroxidase–conjugated Abs were purchased from Santa Cruz Biotechnology (Santa Cruz, CA, USA) (1 : 1000 dilution).

### 2.10. Data and Statistical Analyses

The 3D image overlays were visualized with the Leica Application Suite (LAS) Advanced Fluorescence Lite software (LAS AF Lite, 2.4.1 build 6384, Leica). The ImageJ software (National Institutes of Health, Bethesda, MD, USA) was used to analyze the immunohistochemistry results. Analyses of plaque distributions were transcribed manually into computer-acceptable format by keeping research colleagues blind. In the case of single mean comparison, Student's *t* test was performed to analyze the data. In case of multiple mean comparisons, ANOVA followed by Newman–Keuls posttest, or two-way repeated-measures ANOVA, followed by Bonferroni tests, was used. Data are expressed as means ± standard deviations of the means (SD). *P* value <0.05 was considered statistically significant (Prism version 6.0 software (GraphPad), USA).

## 3. Results

### 3.1. The Expression of NLRC3 Is Downregulated in Brain Tissues of APP/PS1 Mice

**Figure 1:**
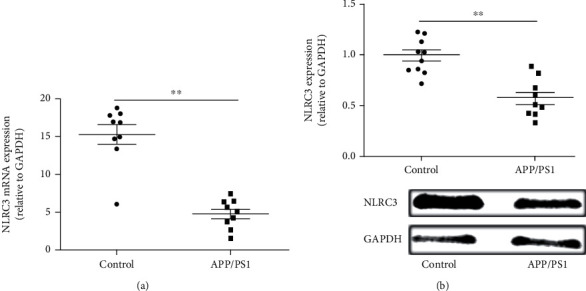
NLRC3 is downregulated in brain tissues of APP/PS1 mice. The expression of NLRC3(a) mRNA and (b) protein was examined in brain tissues in both groups by qPCR and western blotting. Data are expressed as the mean ± standard error of the mean (*n* = 8). An unpaired *t*-test was used for this analysis. ^∗∗^*P* < 0.01.

All mice were genetically characterized. We further investigated the expression of NLRC3 in the hippocampus of mice. q-PCR showed that the mRNA of NLRC3 was detected in the hippocampus of both control and APP/PS1 mice. Compared with the control group, the expression of NLRC3 mRNA in the hippocampus of APP/PS1 mice decreased significantly. The trend of the protein expression is consistent with mRNA of NLRC3. The expression of the NLRC3 protein was detected in the hippocampus of both control and APP/PS1 mice, and the expression of the NLRC3 protein was significantly decreased in the hippocampus of the APP/PS1 group (Figures 1(a) and 1(b)). That was to say, total NLRC3 mRNA and protein levels were significantly decreased in the brains of APP/PS1 mice compared to controls.

### 3.2. The Overexpression of NLRC3 Improved the Learning and Memory Ability of APP/PS1 Mice

**Figure 2:**
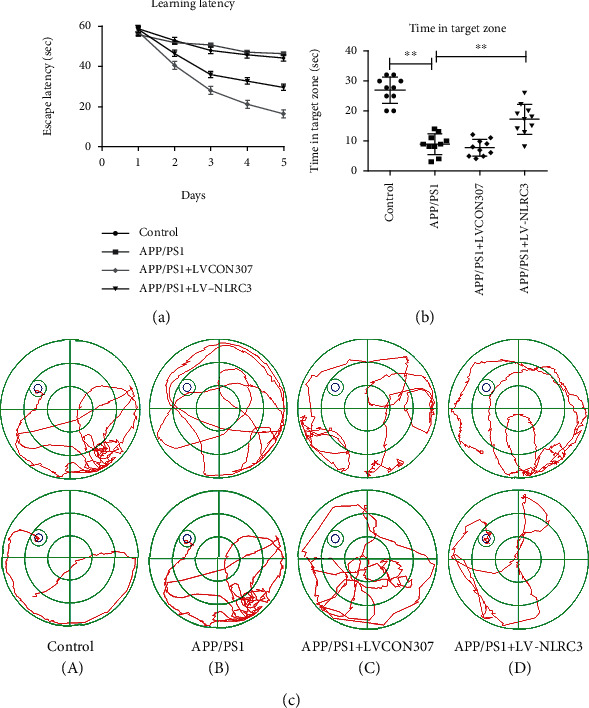
NLRC3 improves the learning and memory ability of APP/PS1 mice. (a) Learning latency were obtained and recorded from each group. (b) The retention time in the target area in each group. (c) The Morris water maze test was performed in each group. Data are expressed as the mean ± standard error of the mean (*n* = 10). An unpaired *t*-test was used for this analysis. ^∗∗^*P* < 0.01.

As the expression of NLRC3 was decreased in the brains of APP/PS1 mice, we hypothesized that the overexpression of NLRC3 may alleviate the progression of AD. To answer this question, in the current study, mice were divided into four groups with different treatments (control, APP/PS1 group, APP/PS1 + LVCON307, and APP/PS1 + LV-NLRC3 group). The spatial learning and memory ability of mice were assessed with the Morris water maze. Mice in the APP/PS1 group had a significant attenuation of learning and memory ability compared to the control group (Figures 2(a)–2(c)). There was no significant difference in the time taken to reach the platform between the four groups on day 1 (*P* > 0.05). However, the time to reach the platform was significantly shorter in the APP/PS1 + LV-NLRC3 group than in the APP/PS1 group on day 2 (41.60 ± 0.49 s vs. 53.40 ± 0.40s, respectively, *P* < 0.001), day 3 (28.30 ± 2.07 s vs. 49.60 ± 1.27 s, respectively, *P* < 0.001), day 4 (21.10 ± 1.41 s vs. 46.00 ± 1.16 s, respectively, *P* < 0.001), and day 5 (16.60 ± 0.97 s vs. 47.80 ± 1.74 s, respectively, *P* < 0.001) (Figure 2(a)). There was no significant difference between APP/PS1 and APP/PS1 + LVCON307 groups. We then evaluated the memory ability at the same time and found that the retention time in the target area was significantly higher in the APP/PS1 + LV-NLRC3 group (20.80 ± 2.34 s) than in the APP/PS1 group (9.00 ± 1.07 s, *P* < 0.001) in the probe trial (Figure 2(b)). Our data suggest that APP/PS1 mice displayed AD-like symptoms with impaired learning and memory ability, and this defect can be partially rescued in consistent with the overexpression of NLRC3 in the APP/PS1 + LV-NLRC3 group (Figure 2(c)).

### 3.3. The Overexpression of NLRC3 Inhibited the Deposition of A*β*, Decreased the Activation of Glial Cells, and Reversed the Degeneration of Neurons in APP/PS1 Mice. NLRC3 Colocalized with NeuN

**Figure 3:**
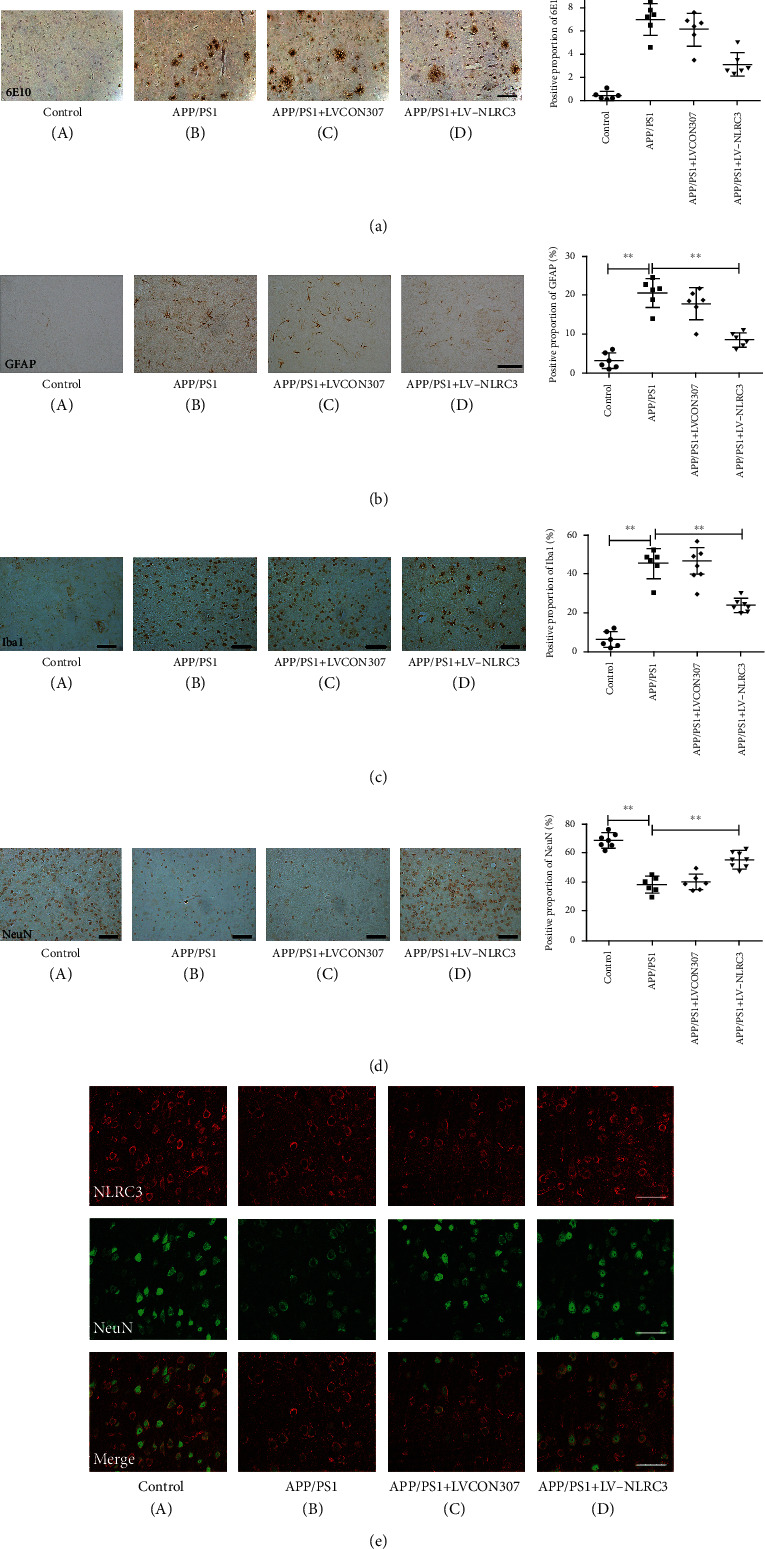
NLRC3 inhibits the deposition of A*β*, decreases the activation of glial cells, and reverses the degeneration of neurons in APP/PS1 mice. NLRC3 colocalize with NeuN. (a) Immunohistochemistry and quantification of 6E10 in brain tissues (magnification ×200; *n* = 6). (b) Immunohistochemistry and quantification of GFAP in brain tissues (magnification ×200; *n* = 6). (c) Immunohistochemistry and quantification of Iba1 in brain tissues (magnification ×200; *n* = 6). (d) Immunohistochemistry and quantification of NeuN in brain tissues (magnification ×200; *n* = 6). (e) Immunofluorescence and laser scanning confocal microscopy to measure the expressions of NeuN with NLRC3 in brain tissues of each group. NLRC3 and NeuN were labeled by Red fluorescence (Cy3) and green fluorescent proteins, respectively. Data are expressed as the mean ± standard error of the mean (*n* = 6). ANOVA and Student–Newman–Keuls test were used for these analyses. ^∗∗^*P* < 0.01.

The APP/PS1 model is known to produce elevated levels of A*β* and develop AD-like phenotypes by expressing mutant APP and PS1. A*β*-dependent inflammation is considered to reflect the extent of injury and toxicity in AD. With 6E10 staining, we found that there was little deposition of A*β* in the control group. In APP/PS1 and APP/PS1 + LVCON307 groups, scattered or agglomerated deposition of A*β* could be seen. After the overexpression of NLRC3, deposition of A*β* in the APP/PS1 + LV-NLRC3 group decreased significantly compared with that in APP/PS1 and APP/PS1 + LVCON307 groups (Figure 3(a)).

Uncontrolled and overactivated microglia and astrocytes are prominent sources of proinflammatory factors such as IL-1*β*, IL-6, and TNF-a, which are neurotoxic [27]. GFAP is a kind of intermediate filament protein, which mainly exists in astrocytes, and is a specific marker of astrocyte activation in the nervous system. Iba1 plays an important role in regulating some immune and pathophysiological functions of microglia and can be used as a specific marker of microglia activation. To reveal the changes of astrocytes and microglial, we performed immunostaining using GFAP and Iba1 antibodies. In brain tissues of the control group, there were low expression of both GFAP and Iba1. In contrast, there were increased amount of GFAP and IBA1 detected in brain tissues of both APP/PS1 and APP/PS1 + LVCON307 groups. However, the expression of GFAP and Iba1 in brain tissues of the APP/PS1 + LV-NLRC3 group was significantly reduced compared with that in APP/PS1 and APP/PS1 + LVCON307 groups (Figures 3(b) and 3(c)).

One pathological feature of the AD is neuronal loss. Many studies have suggested that the pronounced decline of neurons in the brain contribute to the deterioration of the cognitive function. In this study, NeuN was analyzed to evaluate neuron damage. NLRC3 and NeuN were labeled by Red fluorescence (Cy3) and green fluorescent proteins, respectively. The two fluorescent densities were analyzed separately, or merged using Adobe Photoshop Version to identify colocation of NLRC3 and NeuN. NLRC3 localized at neurons. Both the red and green fluorescence were strong in brain tissues of the control group indicating high levels of NLRC3 and NeuN. The expressions of NLRC3 and NeuN in the brains of mice in APP/PS1 and APP/PS1 + LVCON307 groups were significantly downregulated compared to that in the control group. Compared with the APP/PS1 and APP/PS1 + LVCON307 groups, the red and green fluorescence of the APP/PS1 + LV-NLRC3 group was significantly stronger, but still weaker than the control group suggesting that the expression of NLRC3 was negatively associated with degree of neuron-degeneration progression. NLRC3 was colocalization with NeuN (Figures 3(d) and 3(e)).

### 3.4. The Overexpression of NLRC3 Attenuates the Activation of PI3K in the Brains of APP/PS1 Mice

**Figure 4:**
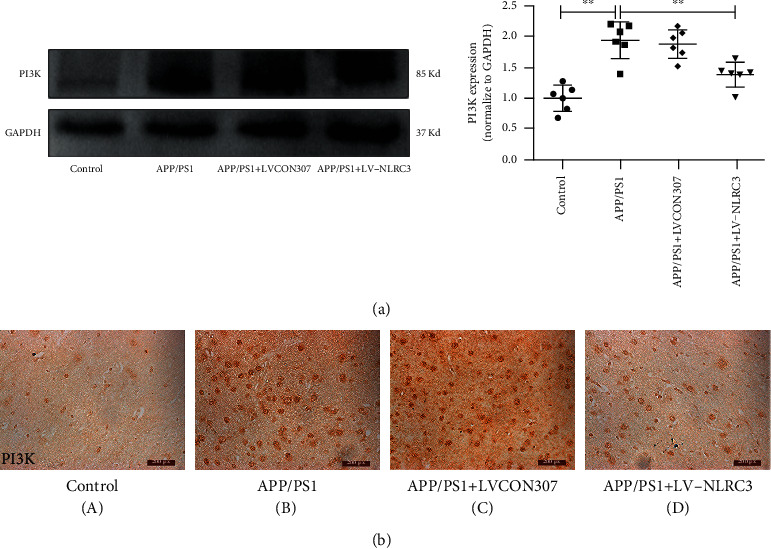
NLRC3 attenuates the activation of PI3K in the brains of APP/PS1 mice. (a) The expression of the PI3K protein was determined in both groups by western blotting (*n* = 6). (b) PI3K immunohistochemistry was performed in brain tissues (magnification ×200). Data were expressed as the mean ± standard error of the mean (*n* = 6). An unpaired *t*-test was used for this analysis. ^∗∗^*P* < 0.01.

Studies have confirmed that PI3K and its downstream signaling pathway play an important role in the occurrence and development of AD. Studies have also shown that NLRC3 can regulate the expression of PI3K in different diseases and NLRC3 can interact with PI3K. Then, the next question is NLRC3 related to PI3K in AD? We found that contrary to the trend of NLRC3, the PI3K expression was significantly upregulated in the hippocampus of APP/PS1 mice by WB. There was no significant difference in the PI3K expression between APP/PS1 and APP/PS1 + LVCON307 groups. Following the overexpression of NLRC3, the activation of PI3K was significantly inhibited in the APP/PS1 + LV-NLRC3 group (*P* < 0.05; Figure 4(a)). Immunohistochemistry also indicated that the expression of PI3K localized in neurons was significantly higher in the APP/PS1 group than that in the control group. The activation of PI3K was inhibited after the overexpression of NLRC3 in the APP/PS1 + LV-NLRC3 group (Figure 4(b)).

### 3.5. NLRC3 Reverses Senile Plaque Deposition, Neuronal Loss, and PI3K Activation Induced by A*β* in Hippocampal Slices

**Figure 5:**
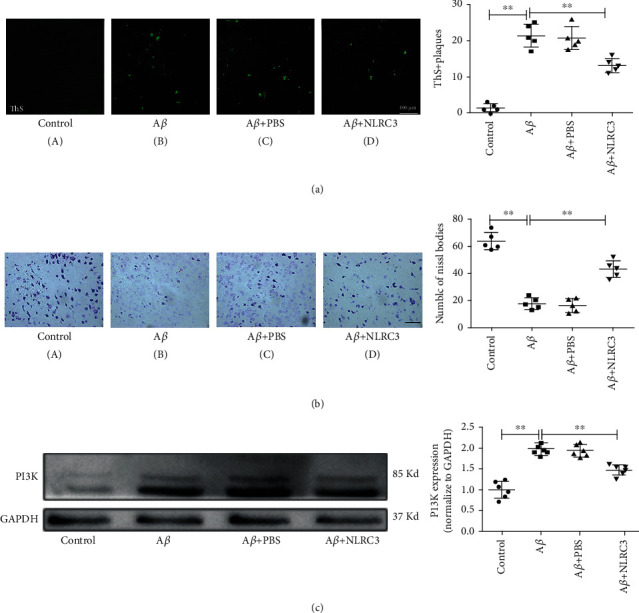
NLRC3 reverses senile plaque deposition, neuronal loss, and PI3K activation induced by A*β* in hippocampal slice. (a) ThS staining was applied to analyze the number and area of the senile plaques in mice hippocampal slices (magnification ×100, *n* = 5). (b) Nissl bodies were analyzed to evaluate neuron damage with Nissl staining in mice hippocampal slices (magnification ×100, *n* = 5). (c) The expression of the PI3K protein was determined in mice hippocampal slices by western blotting (*n* = 6).

ThS-negative diffuse senile plaques are characterized as a pathological sign of AD. ThS staining was applied to analyze the number and area of the senile plaques in mice hippocampal slices. No senile plaque deposition was observed in the control group. In A*β* and A*β* + PBS groups, the senile plaques were distributed in intracellular and extracellular spaces in spherical or irregular shapes of different sizes. Hippocampal slices from the A*β* + NLRC3 group exhibited a lower senile plaques burden compared with both A*β* and A*β* + PBS groups (Figure 5(a)).

Nissl bodies were also analyzed to evaluate neuron damage. With Nissl staining, we detected that the neurons were of distinct and regular structure, and the neuronal density was clearly higher in the control group. In contrast, hippocampal slices from that A*β* and A*β* + PBS groups revealed injuries including remarkable neuron loss, karyopycnosis, and disappearance of Nissl bodies. After administration of the exogenous NLRC3 protein, the neuronal density was increased while the vacuolization of endochylema and karyopycnosis was decreased significantly in the A*β* + NLRC3 group compared to both A*β* and A*β* + PBS groups (Figure 5(b)).

We further examined the expression of PI3K in hippocampal slices of each group. The results showed that the trend of PI3K detected by WB in hippocampal slices were consistent with that in vivo. In the A*β* group, PI3K was significantly upregulated compared with that in the control group. After administration of the exogenous NLRC3 protein, the activation of PI3K was significantly inhibited in the A*β* + NLRC3 group compared to both A*β* and A*β* + PBS groups (Figure 5(c)).

### 3.6. PI3K Agitation Partially Reverses the Protective Effect of NLRC3 on the Hippocampus of Mice Exposed to A*β*

The deposition of senile plaques, dysfunction of glial cells, and loss of neurons in AD are related to the activation of PI3K. The results detailed above suggest that the NLRC3 overexpression could reduce plaque deposition and neuron loss in the hippocampus of AD model mice, decrease the abnormal activation of glial cells, and inhibit the activation of PI3K. Next, we explored whether activation of PI3K would reverse the protective effect of NLRC3 on the hippocampus of mice exposed to A*β*.

**Figure 6:**
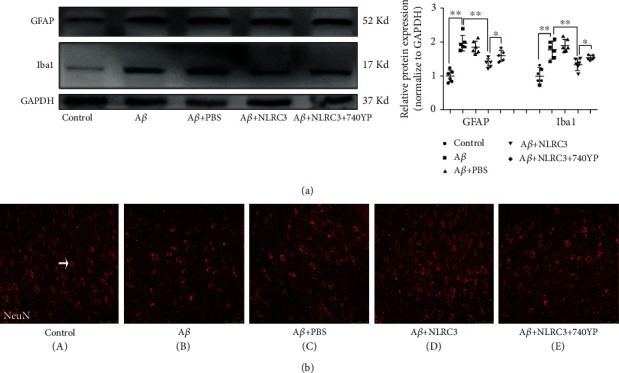
PI3K agitation reverses the protective effect of NLRC3 in the hippocampus of mice exposed to A*β*. (a) Protein expression of GFAP and Iba1 was determined by western blotting in mice hippocampal slices. Data were expressed as the mean ± standard error of the mean (*n* = 6). (b) Immunofluorescence was performed to assess the expression of NeuN in mice hippocampal slices (magnification ×200, *n* = 6). ^∗^*P* < 0.05, ^∗∗^*P* < 0.01.

In this experiment, elevations of GFAP and Iba-1 in response to A*β* were observed in the A*β* group. In the A*β* + NLRC3 group, NLRC3 could inhibit the elevation of GFAP and Iba-1 induced by A*β*. In the A*β* + NLRC3+ 740YP group, PI3K activation with 740YP increased the expression of GFAP and Iba-1 in the hippocampus with the exogenous NLRC3 protein. That was to say, PI3K activation with 740YP could antagonize the effect of NLRC3 to some extent (*P* < 0.05, Figure 6(a)).

To explore the effect of PI3K activation on neurons after the NLRC3 administration to hippocampal slice, we further investigated the information of neuron loss under different interventions using immunofluorescence. The results showed that the expression of NeuN (red fluorescent) in the A*β* group was significantly lower than that in the control group. After the exogenous NLRC3 administration, the expression of NeuN in the A*β* + NLRC3 group was significantly higher than that in the A*β* group, but still lower than that in the control group. However, after administration of 740YP (PI3K agonist), the expression of NeuN in the A*β* + NLRC3+ 740YP group was higher than that in the A*β* + NLRC3 group, indicative of partial reversal (Figure 6(b)).

**Figure 7:**
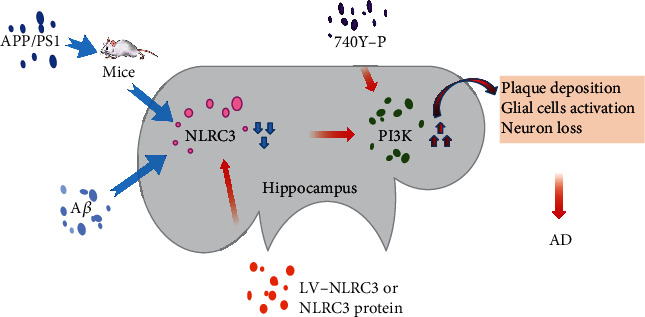
Proposed pathway of NLRC3 mediation which contributes to AD. A*β* and APP/PS1 downregulated the expression of NLRC3 and consequently increased the PI3K expression and activation. PI3K-related functional proteins contributed to A*β* and APP/PS1-induced plaque deposition, glial cell activation, and neuron loss in AD. The NLRC3 overexpression inhibited the activation of PI3K; on the contrary, this effect of NLRC3 could be partially reversed by 740Y-P (PI3K agonist).

Collectively, experiments in this study confirm that the downregulation of NLRC3 can lead to the activation of PI3K, resulting in abnormal plaque deposition, glial cell activation, and neuron loss during AD. The NLRC3 overexpression inhibited the activation of PI3K; however, this effect of NLRC3 could be partially reversed by 740Y-P (PI3K agonist) (Figure 7). That is to say, NLRC3 delays the progression of AD in APP/PS1 mice via inhibition of PI3K activation.

## 4. Discussion

Our study demonstrates the novel role of NLRC3 in a mouse model of AD and thus its potential involvement in AD pathogenesis. The expression of NLRC3 decreased in brain tissues of APP/PS1 mice compared to control mice. Additionally, we show that function augmentation of NLRC3 can effectively improve spatial learning and memory function and increase NeuN expression, while inhibiting plaque deposition, neuronal loss, and PI3K activation in APP/PS1 mice.

AD is the most common cause of dementia in the elderly and has insidious onset, chronic progression, and long duration. Although, to date, several independent hypotheses have been proposed to explain the disease, pharmacological treatments of AD remain limited, and the prognosis is still poor. Thus, further studies are needed to target mechanisms of AD and develop specific drugs. According to this study, the spatial learning and memory function were significantly decreased in APP/PS1 mice compared to control mice. In the present study of AD model mice, neurons were injured seriously, plaque deposition were increased, and PI3K signals were activated, which were almost consistent with the results of previous study [28].

The activation of the PI3K pathway plays an important role in the occurrence and development of AD. Many studies have found that PI3K activation can lead to abnormal activation and functional changes of glial cells. In AD, the activation of glial cells leads to a series of adverse reactions, such as neuroinflammation, and oxidative stress. Many signal factors, including PI3K and Akt, play important roles in glial cell activation and neuritis [29]. GSK-3b, a downstream regulator of PI3K/Akt, is one of the important kinase involved in the hyperphosphorylation of tau protein. The GSK-3b overexpression in transgenic mice represents tau hyperphosphorylation, neuroinflammation, and cognitive dysfunctions. Melatonin ameliorates A*β*-induced memory deficits, tau hyperphosphorylation, and neurodegeneration via the PI3/Akt/GSk3*β* pathway in the mouse hippocampus [30]. It has been reported that caspase-3 directly and specifically regulates the development of A*β* and tau neuropathology and next demonstrates that this effect is mediated by the GSK3*β* kinase pathway via a caspase-3-dependent cleavage of Akt [31]. In bacterial LPS-induced neuritis, P13K-Akt can be activated by binding to its specific receptor and interact with multiple upstream molecules to regulate glial cell-induced neurodegeneration [32].

Increasing evidence suggests that inflammation is a prominent feature of AD and correlates to its pathogenesis. Microglia and astrocytes are main mediators of inflammation in the brain. In a healthy brain, these cells release molecules that help maintain a normal brain function. In AD, they become dysregulated, causing detrimental effects such as neuroinflammation that can promote the development and progression of neurodegeneration [33]. Activation of microglia and astrocytes initiates the inflammatory cascade that leads to the release of potentially neurotoxic cytokines which are suggested to play a decisive role in the pathogenesis of AD [34]. Increasing evidence reveals that dysfunction of the PI3K/Akt signaling pathway is closely related to several features of the pathology of AD, including neuroinflammation mediated by gliocyte activation, A*β* production, and neuron loss [35]. Previous studies identified therapies that regulate the PI3K/Akt signaling pathway exert cognitive deficits improving effects in experimental models of AD via suppressing neuroinflammation, amyloid pathology, and synaptic dysfunction [36]. Our examination demonstrated that in the brain tissues of APP/PS1 mice, the activation of glial cells, as assessed by Iba1 and GFAP immunoreactivity, was increased together with the activation of PI3K. On the other hand, the expression of NLRC3 and NeuN and the number of Nissl bodies decreased markedly in the brain tissues of APP/PS1 mice compared with control mice. The overexpression of NLRC3 significantly inhibited the activation of PI3K and reversed the increasing of GFAP and Iba1, as well as improving the contents of NeuN and Nissl bodies. This suggests that NLRC3 could alleviate neurodegeneration in APP/PS1 mice.

In an AD brain, A*β* plaques surrounding astrocytes and microglia can secrete inflammatory mediators to regulate neuroinflammation. IL-1*β*, IL-6, and TNF*α* are involved in the initiation and progression of AD by deregulating A*β*-mediated inflammation and APP metabolism. We performed LV-NLRC3 administration studies to identify the contribution of NLRC3 in APP/PS mice during A*β* formation. As expected, APP/PS mice which received LV-NLRC3 had significantly lower A*β* burden compared with APP/PS mice that did not receive LV-NLRC3. However, APP/PS mice which received LV-NLRC3 still had significantly increased A*β* burden compared with control mice. These results supported the notion that disruption of NLRC3 may increase A*β* generation. In a AOM-DSS inflammation-induced colorectal cancer model, defective inflammasome activation leads to loss of epithelial integrity, massive leukocyte infiltration, and increased chemokines expression in NLRC3−/− mice, NLRC3 seems important for the suppression of inflammation [20, 37]. On the other hand, Shiau CE et al. demonstrate that NLRC3-like can alleviate unwarranted inflammation in microglia precursor cells and exert a neuroprotective effect through decreasing proinflammaory factors such as IL-1*β* and IL-8 during systemic inflammation [38]. In line with these findings, our data demonstrate lower expressions of GFAP and Iba1 in APP/PS1 + LV-NLRC3 mice than that in APP/PS1 mice. These results might further confirm the hypothesis that NLRC3 provides a neuronal protection effect in APP/PS1 mice, as it can regulate the functions of glial cells and alleviate neuron loss by inhibiting the activation of the PI3K signaling pathway.

However, research into NLRC3 and its role in AD pathogenesis remains in its infancy. PI3K activity of astrocytes is highly associated with neurodegenerative diseases. Increased expression of PI3K has been found in the immediate vicinity of extracellular A*β* deposition in patients with dementia and in an AD mice model. In astrocytes, A*β* elicits the production of ROS and nitric oxide (NO), which can activate PI3K and its downstream signals, thereby leading to AD-related events in the brain including neurodegeneration and inflammation [39]. Moreover, functional inhibition of PI3K abrogated the production of A*β* plaques, IL-1*β*, IL-6, and TNF. We proposed that A*β* attenuates the expression of NLRC3 and subsequently increases the activation of the PI3K signaling pathway, thereby leading to production of proinflammatory cytokines from astrocytes and microglia. In our study, the expression of PI3K in APP/PS1 mice was significantly higher compared with that in control mice, while in contrast NLRC3 was lower. Treatment with NLRC3 significantly reversed the increase of PI3K in APP/PS1 mice and A*β* hippocampal slices. NLRC3 may lessen the PI3K activity and its downstream signaling pathways, which are involved in inflammation and AD. Our results indicate that NLRC3 may play an important role through PI3K signaling pathways in AD progression. However, the detailed molecular mechanisms by which NLRC3 regulates AD progression need further investigation.

Finally, behavioral analysis demonstrated that NLRC3 impeded AD progression and improved neuropsychiatric signs, positively affecting cognition and spatial learning and memory.

Collectively, our data suggest that NLRC3 is involved in the progression of AD and hence may provide a novel therapeutic strategy and biomarker for AD. However, this study may have several drawbacks. We only used the hippocampal slice culture in vitro to study the mechanism. No specific studies have used cell lines of the nervous system. Therefore, it was not possible to explore the mechanisms at a deeper level. Moreover, we did not study brain slices other than of the hippocampal slices, and whether these result would be replicated in other areas of the brain remains to be verified. Finally, we only established the NLRC3 overexpression mice model without also examining a knockout group.

Our preliminary study found that NLRC3 and neurons have colocalization phenomena. Next, we plan to culture neurons and explore the protective effect of NLRC3 on neurons through cell experiments. We will establish AD models that overexpress or knock out NLRC3 and directly intervene in the PI3K pathway to support our current conclusions.

## 5. Conclusion

In conclusion, we provide experimental evidence to support the critical role of NLRC3 in the progression of AD. The molecular mechanisms we establish may provide important information for elucidating the pathogenesis of AD and aid in the development of therapeutic interventions for AD.

## Data Availability

All raw data used in this manuscript are available on request.

## Abbreviations

AD: Alzheimer's disease
A*β*: Amyloid beta
NFTs: Neurofibrillary tangles
CNS: Central nervous system
LPS: Lipopolysaccharide
NLRs: Nucleotide-binding domain, leucine-rich repeat containing receptors
NLRC3: Nucleotide-oligomerization domain (NOD)-like receptor subfamily C3
PI3K: Phosphoinositide 3-kinase
ROS: Reactive oxygen species
APP/PS1: APPswe/PS1-dE9 (amyloid precursor protein/presenilin protein 1
LV-NLRC3: Lentivirus NLRC3
LVCON307: Negative control lentivirus
TU: Transducing units
SLM: Spatial learning and memory
DAB: Diamino-benzidine
GFAP: Glial fibrillary acidic protein
NeuN: Neuron-specific nuclear protein
ThS: Thioflavin-S fluorescence
NO: Nitric oxide
RT-qPCR: Real-time quantitative polymerase chain reaction.

## Supplementary Materials

Supplementary Materials Figure S1. PCR genetic identification of genetically altered mouse model of AD. Figure S2. Validation of lentivirus transfection efficiency. (a) qPCR and (b) WB showing that the level of NLRC3 mRNA and protein increased significantly in the transfected group (data were expressed as the mean ± standard error of the mean, *n* = 6). ^∗∗^*P* < 0.01.
