# Supplementary material for: NLRC3 Delays the Progression of AD in APP/PS1 Mice via Inhibiting PI3K Activation
Source: Oxid Med Cell Longev. 2020 Dec 24;2020:5328031. doi: 10.1155/2020/5328031 (PMC7775163; doi:10.1155/2020/5328031)
Supplement: Supplementary Materials — Figure S1. PCR genetic identification of genetically altered mouse model of AD. Figure S2. Validation of lentivirus transfection efficiency. (a) qPCR and (b) WB showing that the level of NLRC3 mRNA and protein increased significantly in the transfected group (data were expressed as the mean ± standard error of the mean, n = 6). ∗∗P < 0.01. [file 5328031.f1.pdf]

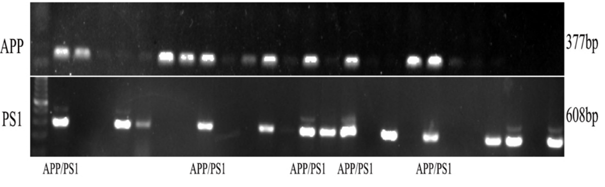
APP
377bp
PS1
608bp
APP/PS1
APP/PS1
APP/PS1
APP/PS1
APP/PS1
